# Free Radical Scavenging Principles of *Salvia reuterana* Boiss. Aerial Parts

**DOI:** 10.22037/ijpr.2020.1101055

**Published:** 2020

**Authors:** Younes Panahi, Mostafa Ghanei, Abbas Hadjiakhoondi, Sheyda Ahmadi-Koulaei, Mohammad-Reza Delnavazi

**Affiliations:** a *Chemical Injuries Research Center, Systems Biology and Poisonings Institute, Baqiyatallah University of Medical Sciences, Tehran, Iran. *; b *Department of Pharmacognosy, Faculty of Pharmacy, Tehran University of Medical Sciences, Tehran, Iran.*

**Keywords:** Salvia reuterana Boiss, DPPH, Flavonoid, Rosmarinic acid, Essential oil, GC-MS, Benzyl benzoate

## Abstract

*Salvia reuterana* Boiss. is an aromatic perennial plant traditionally used for its anxiolytic and sedative properties. In the present study, various fractions and essential oil of *S. reuterana* aerial parts were investigated to find its free radical scavenging principles. Hydroalcoholic fraction with IC_50_ value of 112.6 ±3.2 μg mL^-1^ in DPPH assay demonstrated the highest free radical scavenging activity and was selected to further phytochemical investigation. RP-18 and Sephadex LH-20 column chromatography of the hydroalcoholic fraction resulted in the isolation and structural elucidation of four phenolic derivatives, including apigenin-7-O-β-D-glucopyranoside (**1**), luteolin-7-O-β-D-glucopyranoside (**2**), rosmarinic acid (**3**), and luteolin (**4**). Isolated compounds showed potent free radical scavenging activities (5.1-34.2 μg mL^-1^), compared with BHT (21.30 ± 1.9 μg mL^-1^). Twenty four compounds were also identified in GC-MS analysis of the plant essential oil, of which benzyl benzoate (26.64%), *n*-hexyl benzoate (22.99%) and *n*-hexyl isovalerate (6.04%) were the main compounds. The results of the present study introduced *S. reuterana* as a valuable source of natural phenolic antioxidants which can be utilized in prevention of oxidative stress related diseases. Moreover, interesting composition of *S. reuterana* essential oil, dominated by non-terpenes compounds (76.17%) especially aromatic derivatives, make it an appropriate candidate for more detailed studies.

## Introduction

Nowadays, the role of free radicals, particularly reactive oxygen species (ROS), have been well recognized in development of many pathological disorders such as cardiovascular diseases, diabetes, cancers, inflammatory, and neurodegenerative diseases (e.g. Alzheimer’s disease, Parkinson’s disease, etc.) (1). So, in recent years plants have received considerable attention as source of potent and safe natural free radical scavengers to prevent oxidative stress related diseases (2).

The genus *Salvia* L., commonly known as Sage, is one of the largest genera in the Lamiaceae family, mainly distributed in temperate and subtropical regions of the world (3). In the flora of Iran this genus is represented by 61 species, including *Salvia reuterana* Bioss. (4). This perennial aromatic plant is known as ‘’Maryam Goli-e Esfahani” in Persian and its flowering aerial parts are traditionally used in some parts of Iran as anti-depressant, as well as for the treatment of gastrointestinal disorders, eye pains and colds (5, 6). Previous pharmacological studies have confirmed the anxiolytic (7), hypnotic (8), antidiabetic (9), antibacterial (10) and antioxidant (11-13) properties of *S. reuterana* aerial parts. In an comparative study, Esmaeili *et al*. reported that methanol extract of *S. reuterana* had the highest DPPH free radical scavenging activity (IC_50_; 15.1 ± 1.00 µg mL^-1^) and ferric reducing power (FRAP value; 0.34 ± 0.01), among the other tested *Salvia* species (11). Methanol extract of the *S. reuterana* flowers has also been reported to possess higher free radical scavenging activity with (IC_50_; 77.6 µg mL^-1^) in comparison with its leave extract (IC_50_; 119.4 µg mL^-1^), in DPPH assay (12).

As a result of phytochemical studies on this plant aerial part, nine labdane diterpenoids with cytotoxic activity against HeLa and MCF-7 cell lines were isolated from its *n*-hexane extract (14, 15). There are also some reports on essential oil composition of *S. reuterana* from different regions of Iran (10, 12, 16, 17). Regarding the results of previous studies on considerable antioxidant activity of this medicinal species, the aim of the present research was the isolation and identification of the compounds involved in free radical scavenging activity of *S. reuterana.*

## Experimental

*Plant material*

The flowering aerial parts of *S. reuterana* were collected on Jun 2015 from the Khor region, Elburz province, Iran. A voucher specimen was deposited under the code 7045-TEH at the Herbarium of Faculty of Pharmacy, Tehran University of Medical Sciences, Tehran, Iran. 

*Extraction and fractionation*

The air dried and grinded plant aerial parts (400 g) were macerated with 80% methanol in water (5 × 2.5 L). The total hydroalcoholic extract (82 g) was then dissolved in 500 mL of methanol-water mixture (8:2) and extracted by enough volumes of *n*-hexane and chloroform, successively, to get the *n*-hexane, chloroform and residual methanol-water (8:2) soluble (hydroalcoholic) main fractions.

*Essential oil extraction*

Hydrodistillation method using Clevenger apparatus was used to essential extraction from 100 g of dried and comminuted plant material for 3 hours. The pale yellowish oil was dried over anhydrous sodium sulphate and kept at 4 °C until analysis.

*DPPH free radical scavenging assay*

Antioxidant activity of the total extract, main fractions and essential oil of plant aerial parts were evaluated by DPPH (2, 2-diphenyl-1-picryl-hydrazyl) free radical scavenging assay method described by Sarker *et al*. (18). Briefly, twofold serial dilutions (1.0 to 3.9×10^-3^ mg mL^-1^) were made from samples, individually (each 2 mL). Two milliliter of freshly prepared DPPH (Sigma) solution (80 µg mL^-1^) was then added to each test tube. After 30 min, absorptions of the solutions were recorded at 517 nm using an Optizen 2120 UV PLUS spectrophotometer. Butylated hydroxy toluene (BHT) (Sigma), a commercial synthetic antioxidant, was also used as positive control. For each sample concentration causing a 50% reduction in absorption of DPPH solution (40 µg mL^-1^) was calculated as IC_50_. The experiment was repeated three times and results were expressed as Mean ± SD.

*Isolation and purification of compounds*

Hydroalcoholic fraction with the highest free radical scavenging activity (Table 1) was subjected to further phytochemical analysis. A portion of this fraction (4 g) was chromatographed on a reversed-phase (RP18, mesh 230-400, Fluka) column and eluted with the gradient mixture of methanol in water (0.5-9.5 to 7:3) to get five fraction (M1-5). Compounds **1** (18 mg) and **2** (43 mg) were isolated from the fraction M3 (320 mg) on a Sephadex LH-20 column (Fluka) eluted by methanol:water (8:2) as solvent system. Colum chromatography of the fraction M4 (147 mg) on a Sephadex LH-20 column with methanol resulted in the isolation of compound **3** (28 mg). Compound **4** (18 mg) was isolated from the fraction M5 (95 mg) through the reversed-phase column chromatography (methanol-water, 8:2). It was more purified on a Sephadex LH-20 column using methanol as eluent. All column chromatographies were monitored using thin layer chromatography (pre-coated silica gel 60 F-254 sheets, Merck) and the fractions giving same spots under 254 and 366 UV wavelengths were combined.

*GC and GC-MS analysis*

The plant essential oil was analyzed on an Agilent 7890B gas chromatograph with a DB-5 column (30 m × 250 μm id, 0.25 μm) connected to an Agilent 5977A mass selective detector (70 eV) under the following conditions; carrier gas: helium (1 mL min^-1^), temperature program: 50 °C for 5 min, 50-280 °C at 10 °C/min, injector temperature: 280 °C, injection volume: 1 μL. A homologous series of *n*-alkanes was also injected in conditions equal to the oil sample in order to calculate retention indices (RI). The compounds were identified by computer matching with the Wiley7n.L and NIST05a.L libraries, as well as by comparison of RIs and mass fragmentation patterns with those published in literature for standard compounds (19). GC-FID analysis of the oil was performed in the same conditions described above, for quantitative purposes.

## Results and Discussion

**Figure 1:**
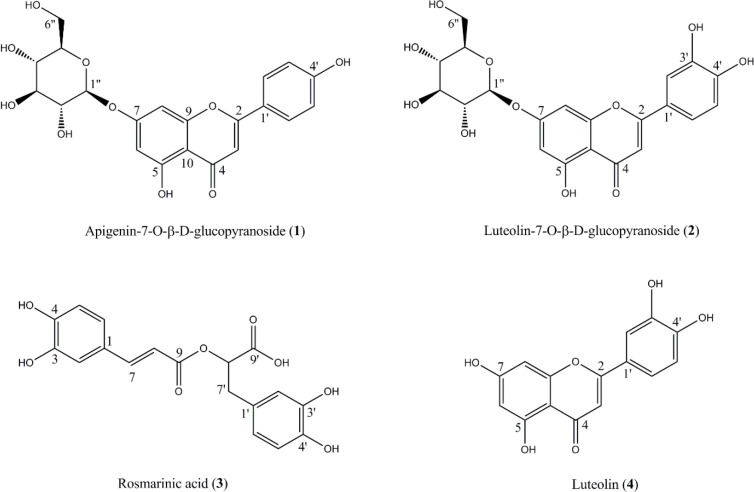
Structures of the isolated compounds (**1**-**4**) from *S. reuterana* aerial parts

In DPPH free radical scavenging assay, hydroalcoholic fraction and essential oil of *S. reuterana* were found to possess higher activity with IC_50_ values of 112.6 ± 3.2 and 246.4 ± 8.1 µg mL^-1^, respectively. Phytochemical analysis of the hydroalcoholic fraction using chromatography on RP-18 and Sephadex LH-20 columns led to the isolation of four phenolic compounds, apigenin-7-O-β-D-glucopyranoside (**1**), luteolin-7-O-β-D-glucopyranoside (**2**), rosmarinic acid (**3**) and luteolin (**4**) (Figure 1). The structures of the isolated compounds (**1**-**4**) were characterized using ^1^H-NMR and ^13^C-NMR spectral analysis (Bruker DRX-500, 500 MHz for ^1^H-NMR and 125 MHz for ^13^C-NMR) and confirmed in accordance with bibliographic data (20-23).

*Spectroscopic data of isolated compounds*

*Apigenin-7-O-β-D-glucopyranoside* (Cosmosiin) (**1**); ^1^H-NMR (DMSO-*d*_6_, 500 MHz): δ 7.94 (2H, *d*, *J=* 8.5 Hz, H-2′,6′), 6.94 (2H, *d*, *J=* 8.5 Hz, H-3′,5′), 6.90 (1H, *s*, H-3), 6.84 (1H, *br s*,H-8), 6.43 (1H, *br s*, H-6), 5.46 (1H, *d*, *J=* 7.0 Hz, H-1″), 3.1-3.9 (6H, H-2″-6″). ^13^C-NMR (DMSO-*d*_6_, 125 MHz): δ 181.93 (C-4), 164.25 (C-2), 162.94 (C-7), 161.84 (C-4′), 161.3 (C-5), 156.94 (C-9), 128.62 (C-2′), 128.50 (C-6′), 120.91 (C-1′), 116.16 (C-3′), 115.87 (C-5′), 105.32 (C-10), 103.25 (C-3), 102.85(C-1″), 99.91 (C-6), 94.60(C-8), 77.18 (C-5″), 76.43 (C-3″), 73.09 (C-2″), 69.56 (C-4″), 60.60 (C-6″) (20).

*Luteolin-7-O-β-D-glucopyranoside* (Cynaroside) (**2**); ^1^H-NMR (DMSO-*d*_6_, 500 MHz): δ 7.45 (1H, *br d*, *J=* 7.0 Hz, H-6′), 7.43 (1H, *br s*, H-2′), 6.94 (1H, *d*, *J=* 7.0 Hz, H-5′), 6.82 (1H, *br s*, H-8), 6.74 (1H, *s*, H-3), 6.46 (1H, *br s*, H-6), 5.08 (1H, *d*, *J=* 7.5 Hz, H-1″), 3.2-3.6 (6H, H-2″-6″). ^13^C-NMR (DMSO-*d*_6_, 125 MHz): δ 182.13 (C-4), 164.82 (C-2), 163.17 (C-7), 161.47 (C-5), 157.25 (C-9), 150.61 (C-4′), 146.15 (C-3′), 121.35 (C-1′), 119.51 (C-6′), 116.34 (C-5′), 113.60 (C-2′), 105.69 (C-10), 103.27 (C-3), 100.24(C-1″), 100.14 (C-6), 95.00 (C-8), 77.34 (C-5″), 76.58 (C-3″), 73.37 (C-2″), 69.88 (C-4″), 60.93 (C-6″) (20, 21).

*Rosmarinic acid* (α-O-caffeoyl-3,4-dihydroxyphenyllactic acid) (**3**); ^1^H-NMR (DMSO-*d*_6_, 500 MHz): δ 7.36 (1H, *d*, *J=* 16.1 Hz, H-7), 7.06 (1H, *br s*, H-2), 6.89 (1H, *br d*, *J=* 6.5, H-6), 6.74 (1H,* d*, *J=* 6.5 Hz, H-5), 6.68 (1H, *br s*, H-2′), 6.60 (1H, *d*, *J=* 7.0 Hz, H-5′), 6.48 (1H, *br d*, *J=* 7.0 Hz, H-6′), 6.17 (1H, *d*, *J=* 16.1 Hz, H-8), 4.86 (1H, *d*, *J=* 8.5, H-8′), 3.02 (1H, *br d*, *J=* 13.5 Hz, H-7′b), 2.75 (1H, *dd*, *J=* 13.5, 11 Hz, H-7′a). ^13^C-NMR (DMSO-*d*_6_, 125 MHz): δ 172.97 (C-9′), 166.34 (C-9), 149.04 (C-4), 146.17 (C-7), 145.06 (C-3), 144.45(C-3′), 143.69 (C-4′), 129.81 (C-1′), 125.25 (C-1), 120.81 (C-6), 119.59 (C-6′), 116.78 (C-2′), 116.05 (C-5), 115.49 (C-5′), 115.04 (C-2), 114.54(C-8), 75.97 (C-8′), 37.17 (C-7′) (22).

*Luteolin* (5,7,3′,4′-Tetrahydroxyflavone) (**4**); ^1^H-NMR (DMSO-*d*_6_, 500 MHz): δ 7.42 (1H, *br d*, *J=* 8 Hz, H-6′), 7.39 (1H, *br s*, H-2′) 6.89 (1H, *d*, *J=* 8 Hz, H-5′), 6.66 (1H, *s*, H-3), 6.44 (1H, *br s*, H-8), 6.18 (1H, *br s*, H-6).^13^C-NMR (DMSO-*d*_6_, 125 MHz): δ 181.61 (C-4), 164.12 (C-2), 163.86 (C-7), 161.45 (C-5), 157.26 (C-9), 149.68 (C-4′), 145.71 (C-3′), 121.46 (C-1′), 118.93 (C-6′), 116.26 (C-5′), 113.32 (C-2′), 103.65 (C-10), 102.82 (C-3), 98.79 (C-6), 93.80 (C-8) (23).

Free radical scavenging activities of the isolated compounds (**1**-**4**) were also assessed by DPPH test. As shown in table 1, among the isolated compounds, luteolin (**4**), rosmarinic acid (**3**) and luteolin-7-O-β-D glucopyranoside (**2**) were found to have a potent free radical scavenging activity with IC_50_ values of 5.1 ± 0.6, 9.6 ± 1.2, 17.3 ± 2.1 µg mL^-1^, higher than positive control, BHT (IC_50_: 21.3 ± 1.9 µg mL^-1^). Thus, these compounds can be assumed as the major free radical scavengers present in *S. reuterana* aerial parts. 

Previously, Farimani and Miran reported the isolation six labdane diterpenoids, namely, sclareol, 6b-hydroxysclareol, 14a-epoxysclareol, 14a-hydroxy-15-chlorosclareol, 14a-hydroxy-15-acetoxysclareol and 6b-hydroxy-14a-epoxysclareol, together with two new diterpenoids, 6β,14α-dihydroxy-15-acetoxysclareol and 14α,15- dihydroxy sclareol from the *n*-hexane extract of *S. reuterana* aerial parts (14, 15). To our knowledge, this is the first report of the isolation and structure elucidation of these phenolic derivatives (**1**-**4**) from the aerial parts of this medicinal species. These compounds, however, have been isolated from various other *Salvia *species (24).

Some biological activities are found in literature for compounds **1**-**4** (25-43). Apigenin-7-O-β-D-glucopyranoside (**1**) has been reported for its anxiolytic (25), insulin mimetic (26), antioxidant (27) and hepatoprotective (28) effects. Luteolin (**4**) and its 7-O-glucopyranoside derivative (**2**) have shown anti-inflammatory (29), chemopreventive (30, 31), antioxidant (32) and α-glucosidase inhibitory (33) activities. Moreover, the results of resent studies reported luteolin as a flavonoid with neuroprotective and anxiolytic effects (34, 35). Accordingly, apigenin-7-O-β-D-glucopyranoside and luteolin with known anxiolytic activity may be involved in anxiolytic properties of *S. reuterana*, which has been previously documented by Rabbani *et al*. (25). Rosmarinic acid (**3**), which has also been reported as a chemotaxonomic marker of the subfamily Nepetoideae (36), is a caffeic acid derivative with a range of health benefit properties such as antioxidant (37), anti-inflammatory (38), antinociceptive (38), hepatoprotective (39) and neuroprotective (40) effects. In 2002, Takeda *et al*. showed that rosmarinic acid (2 mg/kg, i.p.) produces antidepressant-like effect in the forced swimming test in mice (41). Further studies indicated that this antidepressant-like effect is driven at least in part through the proliferation of newborn cells located in the dentate gyrus of the hippocampus (42). Rosmarinic acid has also been found as compound with α-amylase inhibitory activity (43). Combination of α-amylase and α-glucosidase inhibitory effects and insulin mimetic activity, reported from the isolated compounds may be contributed to antidiabetic properties of *S. reuterana*, previously published by Eidi *et al*. (9).

GC-MS analysis of the essential oil resulted in the identification of twenty four compounds, representing the 98.48% of the total oil. The essential oil was rich in non-terpene compounds (76.17%), mainly benzyl benzoate (26.64%), *n*-hexyl benzoate (22.99%) and *n*-hexyl isovalerate (6.04%) (Table 2). Essential oil extracted from *S. reuterana* aerial parts demonstrated notable DPPH free radical scavenging activity (IC_50_: 246.4 ± 5.1 µg mL^-1^). However, the low yield of essential oil extraction (yield: 0.2% (v/w)) attenuates the importance of the plant essential oil in antioxidant properties of *S. reuterana*.

A review on the results of the present study and previous reports shows a variation in essential oil composition of *S. reuterana* aerial parts collected from different regions of Iran (10, 12, 16, 17). In an study by Fattahi *et al*. on essential oil analysis of seven wild population of *S. reuterana* from north and center of Iran, α-gurjunene (5.4-13.7%), β-elemene (4.5-13.9%), germacrene D (2.6-7.2%), spathulenol (1.0-8.0%) and *n*-hexyl acetate (1.2-6.8%) were identified as major compounds (16). Benzyl benzoate, the main compound of our analyzed essential oil sample (26.64%), has been detected in the range of trace to 8.0% in former mentioned study (16). *n-*hexyl benzoate (22.99%), another main compound identified in the present study was not detected by Fattahi *et al*. in their examined essential oils of different *S. reuterana* populations (16). However, *n*-hexyl benzoate has been characterized at high amounts (17.0%) in essential oil of *S. reuterana* flowers, collected from Kashan region, center of Iran (12). Benzyl benzoate and *n-*hexyl benzoate have also been reported in essential oil of *Salvia multicaulis* Vahl aerial parts with relative percentages of 60.3 and 16.7 (44). Differences in climate conditions, as well as possible presence of chemotypes in various *S. reuterana* populations are the factors which could be assumed as responsible for the observed variations in essential oils composition (45). However, a comprehensive study using more advanced chromatographic and spectroscopic techniques is needed for the assessment of variations between essential oil contents of different populations of *S. reuterana*. 

**Figure 2.**
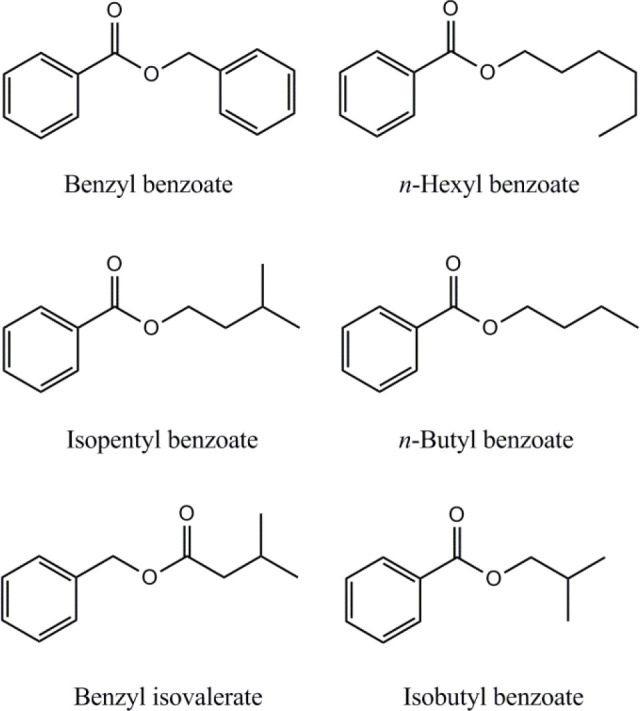
Structures of the aromatic compounds identified in essential oil of* S. reuterana* aerial parts

**Table 1:** Free radical scavenging activities of the essential oil, extract, fractions and isolated compounds from *S. reuterana*

**Samples**	**IC**_50_ ** value (μg mL** ^-1^ **)** ^a^
Essential oil	246.4 ± 5.1
Total extract	187.6 ± 3.5
*n*-Hexane fraction	825.1 ± 12.6
Chloroform fraction	682.5 ± 4.3
Hydroalcoholic fraction	112.6 ± 3.2
Apigenin-7-O-glucoside (**1**)	34.2 ± 1.3
Luteolin-7-O-glucoside (**2**)	17.3 ± 2.1
Rosmarinic acid (**3**)	9.6 ± 1.2
Luteolin (**4**)	5.1 ± 0.6
BHT (Positive control)	21.3 ± 1.9

Note: ^a^ Concentration providing 50% inhibition.

**Table 2:** Chemical composition of the essential oil of *S. reuterana* aerial parts

**No.**	**Compounds** ^a^	**Rt** ^b^	**RI** ^c^	**%**
1	*n*-hexyl acetate	8.27	1009	1.93
2	*n*-butyl isovalerate	8.75	1037	1.05
3	(E)-β-ocimene	8.79	1046	0.68
4	isobutyric acid	10.07	1178	1.07
5	pentyl cyclopropane	10.79	1194	1.22
6	*n*-hexyl 2-methyl butyrate	11.09	1236	1.57
7	*n*-hexyl isovalerate	11.13	1245	6.04
8	isobutyl benzoate	12.11	1329	0.49
9	δ-elemene	12.24	1338	1.31
10	*n*-butyl benzoate	12.56	1356	4.45
11	benzyl isovalerate	12.75	1364	0.83
12	β-elemene	12.79	1392	3.26
13	selin-4,7 (11)-diene	13.07	1412	1.49
14	isopentyl benzoate	13.17	1437	6.40
15	isoledene	13.62	1440	0.82
16	germacrene-D	13.67	1489	0.55
17	δ-selinene	13.73	1497	0.96
18	*n*-hexyl benzoate	14.43	1584	22.99
19	spathulenol	14.54	1588	1.07
20	β-eudesmol	15.16	1654	3.14
21	benzyl benzoate	16.02	1767	26.64
22	sclareol oxide	17.02	1894	1.46
23	manoyl oxide	17.79	1932	0.69
24	sclareol	19.27	2218	8.37
	Monoterpene hydrocarbons			0.68
	Sesquiterpene hydrocarbons			6.90
	Oxygenated sesquiterpenes			4.21
	Diterpenes			10.52
	Non-terpenes			76.17
	Total identified			98.48

Note:^ a^ Identified compounds listed in order of elution from DB-5 column; ^b^ Retention times; ^c^ Retention indices to C_8_–C_24_ *n*-alkanes on DB-5 column.

## Conclusion

The results of present study verify that *S. reuterana* with its potent free radical scavenging flavonoid and caffeic acid derivatives content (**1**-**4**) can be considered as a valuable source of natural phenolic antioxidants. Literature review on biological activities of the isolated compounds also provides some molecular explanations for anxiolytic and antidiabetic properties, previously reported from *S. reuterana*. Moreover, interesting chemical composition of *S. reuterana* essential oils, which is dominated by non-terpenes compounds (76.17%), especially aromatic derivatives, make it an appropriate candidate for further studies.
